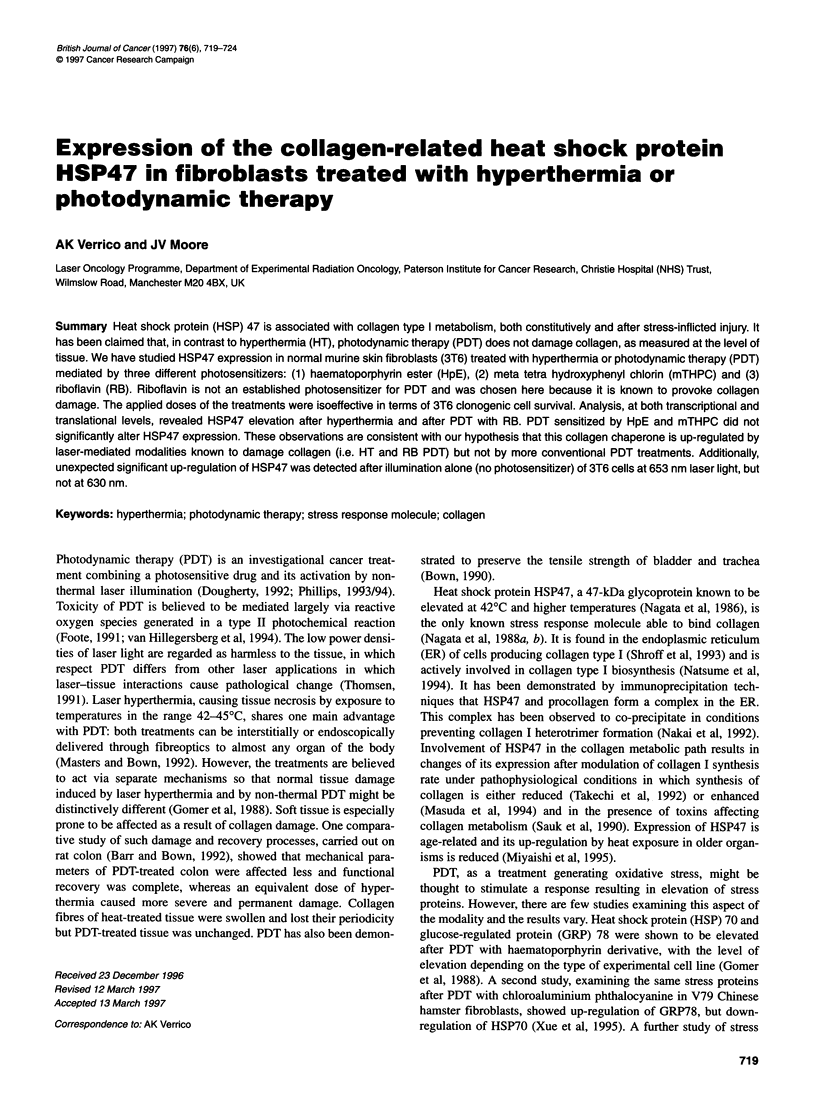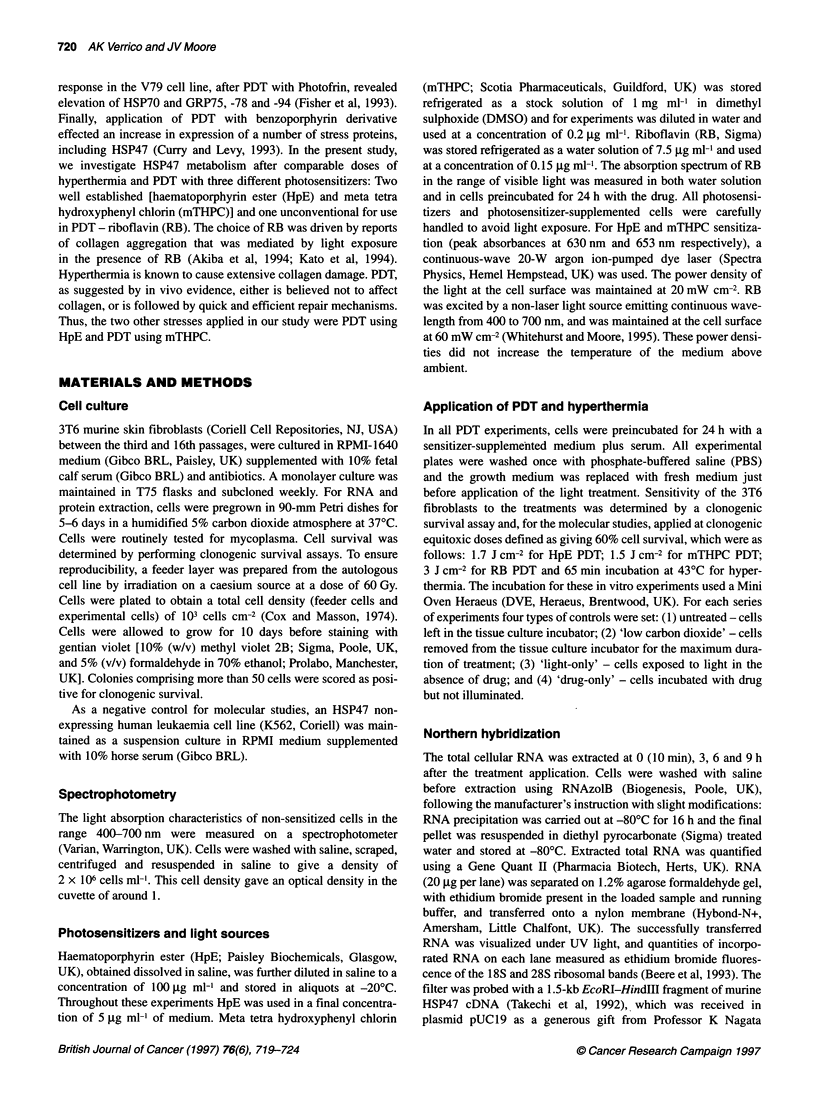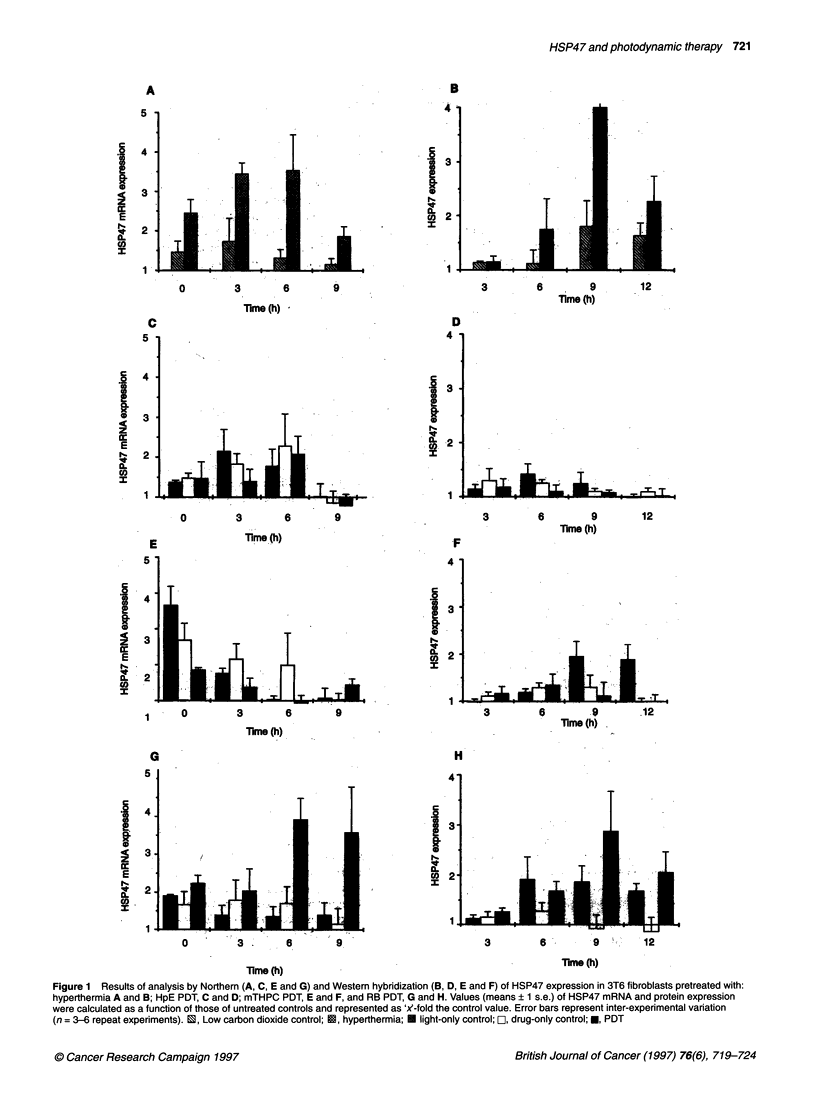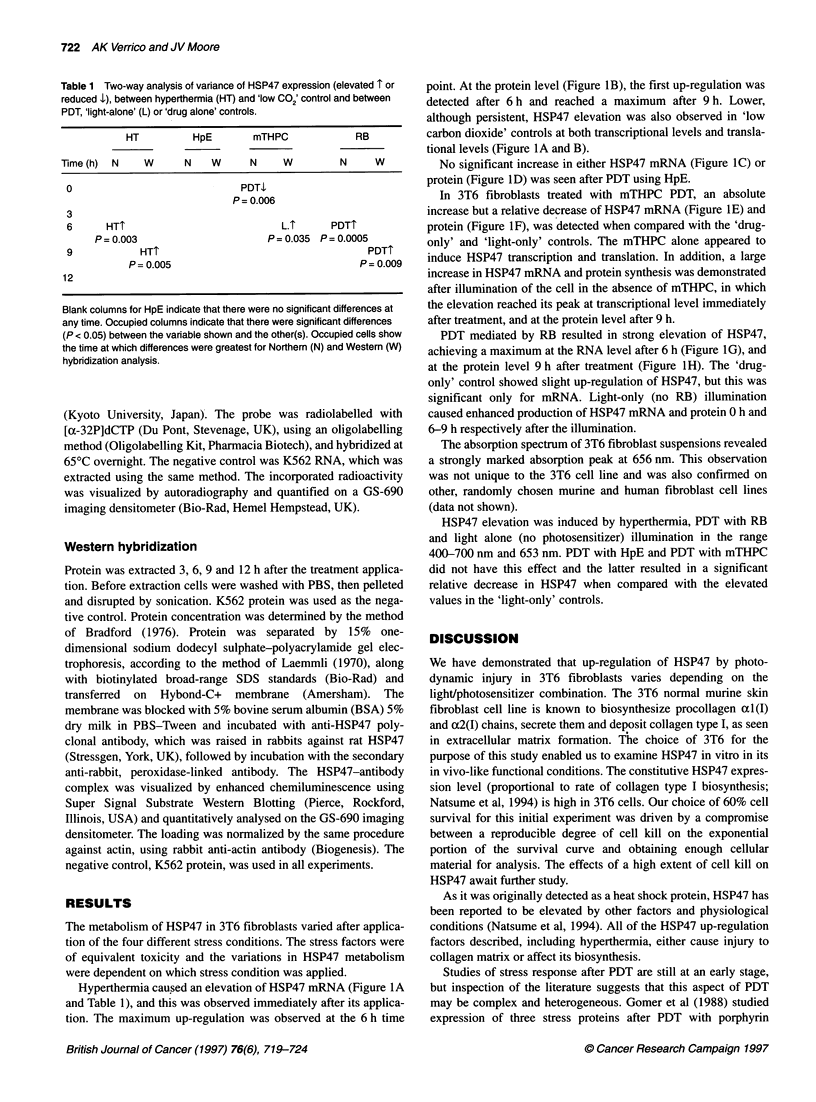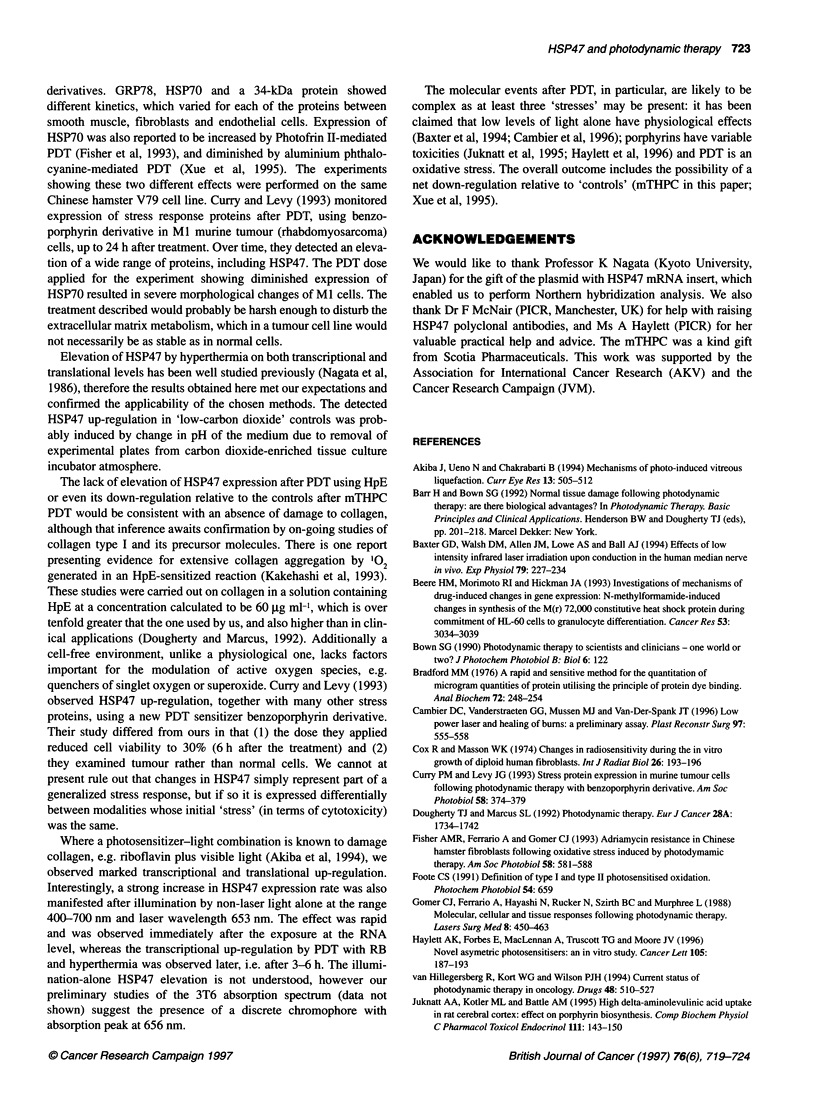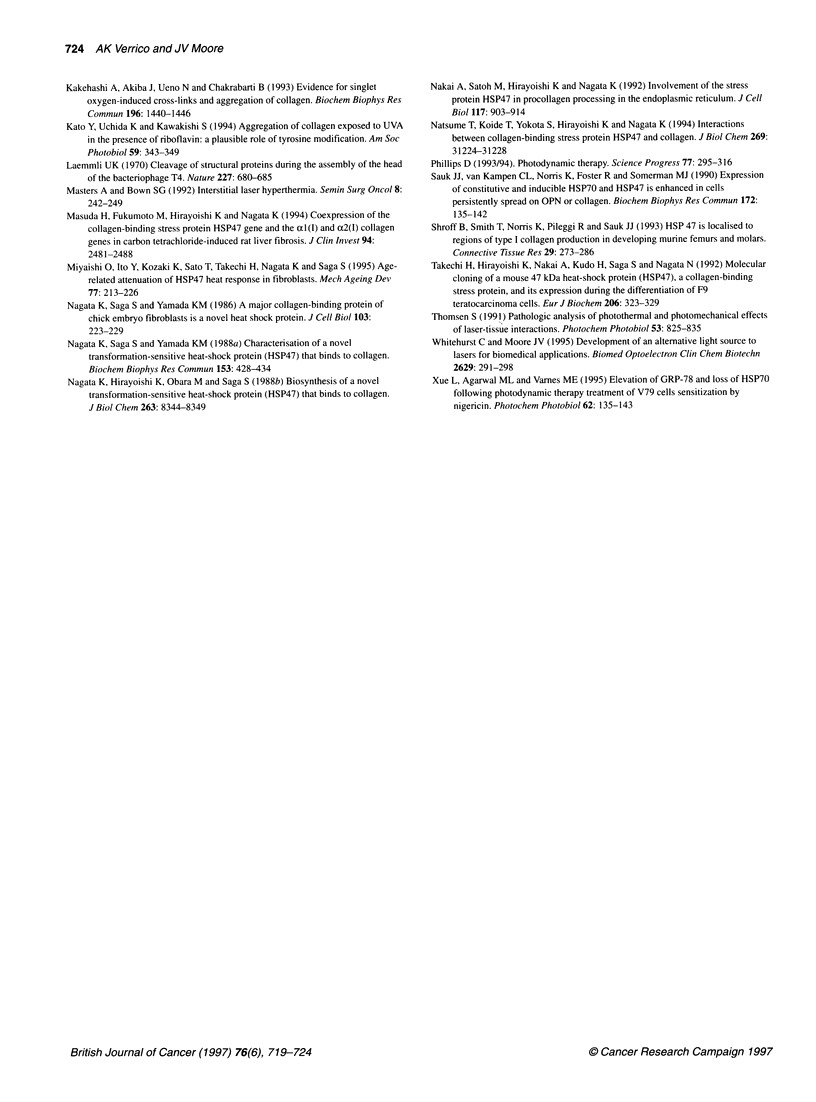# Expression of the collagen-related heat shock protein HSP47 in fibroblasts treated with hyperthermia or photodynamic therapy.

**DOI:** 10.1038/bjc.1997.452

**Published:** 1997

**Authors:** A. K. Verrico, J. V. Moore

**Affiliations:** Department of Experimental Radiation Oncology, Paterson Institute for Cancer Research, Christie Hospital (NHS) Trust, Manchester, UK.

## Abstract

Heat shock protein (HSP) 47 is associated with collagen type I metabolism, both constitutively and after stress-inflicted injury. It has been claimed that, in contrast to hyperthermia (HT), photodynamic therapy (PDT) does not damage collagen, as measured at the level of tissue. We have studied HSP47 expression in normal murine skin fibroblasts (3T6) treated with hyperthermia or photodynamic therapy (PDT) mediated by three different photosensitizers: (1) haematoporphyrin ester (HpE), (2) meta tetra hydroxyphenyl chlorin (mTHPC) and (3) riboflavin (RB). Riboflavin is not an established photosensitizer for PDT and was chosen here because it is known to provoke collagen damage. The applied doses of the treatments were isoeffective in terms of 3T6 clonogenic cell survival. Analysis, at both transcriptional and translational levels, revealed HSP47 elevation after hyperthermia and after PDT with RB. PDT sensitized by HpE and mTHPC did not significantly alter HSP47 expression. These observations are consistent with our hypothesis that this collagen chaperone is up-regulated by laser-mediated modalities known to damage collagen (i.e. HT and RB PDT) but not by more conventional PDT treatments. Additionally, unexpected significant up-regulation of HSP47 was detected after illumination alone (no photosensitizer) of 3T6 cells at 653 nm laser light, but not at 630 nm.


*British Journal of Cancer* (1997) **76**(6), 719–724
© 1997 Cancer Research Campaign

### Expression of the collagen-related heat shock protein HSP47 in fibroblasts treated with hyperthermia or photodynamic therapy

**AK Verrico and JV Moore**

Laser Oncology Programme, Department of Experimental Radiation Oncology, Paterson Institute for Cancer Research, Christie Hospital (NHS) Trust, Wilmslow Road, Manchester M20 4BX, UK

**Summary** Heat shock protein (HSP) 47 is associated with collagen type I metabolism, both constitutively and after stress-inflicted injury. It has been claimed that, in contrast to hyperthermia (HT), photodynamic therapy (PDT) does not damage collagen, as measured at the level of tissue. We have studied HSP47 expression in normal murine skin fibroblasts (3T6) treated with hyperthermia or photodynamic therapy (PDT) mediated by three different photosensitizers: (1) haematoporphyrin ester (HpE), (2) meta tetra hydroxyphenyl chlorin (mTHPC) and (3) riboflavin (RB). Riboflavin is not an established photosensitizer for PDT and was chosen here because it is known to provoke collagen damage. The applied doses of the treatments were isoeffective in terms of 3T6 clonogenic cell survival. Analysis, at both transcriptional and translational levels, revealed HSP47 elevation after hyperthermia and after PDT with RB. PDT sensitized by HpE and mTHPC did not significantly alter HSP47 expression. These observations are consistent with our hypothesis that this collagen chaperone is up-regulated by laser-mediated modalities known to damage collagen (i.e. HT and RB PDT) but not by more conventional PDT treatments. Additionally, unexpected significant up-regulation of HSP47 was detected after illumination alone (no photosensitizer) of 3T6 cells at 653 nm laser light, but not at 630 nm.

**Keywords:** hyperthermia; photodynamic therapy; stress response molecule; collagen

Photodynamic therapy (PDT) is an investigational cancer treatment combining a photosensitive drug and its activation by non-thermal laser illumination (Dougherty, 1992; Phillips, 1993/94). Toxicity of PDT is believed to be mediated largely via reactive oxygen species generated in a type II photochemical reaction (Foote, 1991; van Hillegersberg et al, 1994). The low power densities of laser light are regarded as harmless to the tissue, in which respect PDT differs from other laser applications in which laser–tissue interactions cause pathological change (Thomsen, 1991). Laser hyperthermia, causing tissue necrosis by exposure to temperatures in the range 42–45°C, shares one main advantage with PDT: both treatments can be interstitially or endoscopically delivered through fibreoptics to almost any organ of the body (Masters and Bown, 1992). However, the treatments are believed to act via separate mechanisms so that normal tissue damage induced by laser hyperthermia and by non-thermal PDT might be distinctively different (Gomer et al, 1988). Soft tissue is especially prone to be affected as a result of collagen damage. One comparative study of such damage and recovery processes, carried out on rat colon (Barr and Bown, 1992), showed that mechanical parameters of PDT-treated colon were affected less and functional recovery was complete, whereas an equivalent dose of hyperthermia caused more severe and permanent damage. Collagen fibres of heat-treated tissue were swollen and lost their periodicity but PDT-treated tissue was unchanged. PDT has also been demonstrated to preserve the tensile strength of bladder and trachea (Bown, 1990).

*Received 23 December 1996*
*Revised 12 March 1997*
*Accepted 13 March 1997*

*Correspondence to:* AK Verrico

Heat shock protein HSP47, a 47-kDa glycoprotein known to be elevated at 42°C and higher temperatures (Nagata et al, 1986), is the only known stress response molecule able to bind collagen (Nagata et al, 1988*a, b*). It is found in the endoplasmic reticulum (ER) of cells producing collagen type I (Shroff et al, 1993) and is actively involved in collagen type I biosynthesis (Natsume et al, 1994). It has been demonstrated by immunoprecipitation techniques that HSP47 and procollagen form a complex in the ER. This complex has been observed to co-precipitate in conditions preventing collagen I heterotrimer formation (Nakai et al, 1992). Involvement of HSP47 in the collagen metabolic path results in changes of its expression after modulation of collagen I synthesis rate under pathophysiological conditions in which synthesis of collagen is either reduced (Takechi et al, 1992) or enhanced (Masuda et al, 1994) and in the presence of toxins affecting collagen metabolism (Sauk et al, 1990). Expression of HSP47 is age-related and its up-regulation by heat exposure in older organisms is reduced (Miyaishi et al, 1995).

PDT, as a treatment generating oxidative stress, might be thought to stimulate a response resulting in elevation of stress proteins. However, there are few studies examining this aspect of the modality and the results vary. Heat shock protein (HSP) 70 and glucose-regulated protein (GRP) 78 were shown to be elevated after PDT with haematoporphyrin derivative, with the level of elevation depending on the type of experimental cell line (Gomer et al, 1988). A second study, examining the same stress proteins after PDT with chloroaluminium phthalocyanine in V79 Chinese hamster fibroblasts, showed up-regulation of GRP78, but down-regulation of HSP70 (Xue et al, 1995). A further study of stress response in the V79 cell line, after PDT with Photofrin, revealed elevation of HSP70 and GRP75, -78 and -94 (Fisher et al, 1993). Finally, application of PDT with benzoporphyrin derivative effected an increase in expression of a number of stress proteins, including HSP47 (Curry and Levy, 1993). In the present study, we investigate HSP47 metabolism after comparable doses of hyperthermia and PDT with three different photosensitizers: Two well established [haematoporphyrin ester (HpE) and meta tetra hydroxyphenyl chlorin (mTHPC)] and one unconventional for use in PDT – riboflavin (RB). The choice of RB was driven by reports of collagen aggregation that was mediated by light exposure in the presence of RB (Akiba et al, 1994; Kato et al, 1994). Hyperthermia is known to cause extensive collagen damage. PDT, as suggested by in vivo evidence, either is believed not to affect collagen, or is followed by quick and efficient repair mechanisms. Thus, the two other stresses applied in our study were PDT using HpE and PDT using mTHPC.

### MATERIALS AND METHODS

### Cell culture

3T6 murine skin fibroblasts (Coriell Cell Repositories, NJ, USA) between the third and 16th passages, were cultured in RPMI-1640 medium (Gibco BRL, Paisley, UK) supplemented with 10% fetal calf serum (Gibco BRL) and antibiotics. A monolayer culture was maintained in T75 flasks and subcloned weekly. For RNA and protein extraction, cells were pregrown in 90-mm Petri dishes for 5–6 days in a humidified 5% carbon dioxide atmosphere at 37°C. Cells were routinely tested for mycoplasma. Cell survival was determined by performing clonogenic survival assays. To ensure reproducibility, a feeder layer was prepared from the autologous cell line by irradiation on a caesium source at a dose of 60 Gy. Cells were plated to obtain a total cell density (feeder cells and experimental cells) of $10^3$ cells $cm^{-2}$ (Cox and Masson, 1974). Cells were allowed to grow for 10 days before staining with gentian violet [10% (w/v) methyl violet 2B; Sigma, Poole, UK, and 5% (v/v) formaldehyde in 70% ethanol; Prolabo, Manchester, UK]. Colonies comprising more than 50 cells were scored as positive for clonogenic survival.

As a negative control for molecular studies, an HSP47 non-expressing human leukaemia cell line (K562, Coriell) was maintained as a suspension culture in RPMI medium supplemented with 10% horse serum (Gibco BRL).

### Spectrophotometry

The light absorption characteristics of non-sensitized cells in the range 400–700 nm were measured on a spectrophotometer (Varian, Warrington, UK). Cells were washed with saline, scraped, centrifuged and resuspended in saline to give a density of $2 \times 10^6$ cells $ml^{-1}$. This cell density gave an optical density in the cuvette of around 1.

### Photosensitizers and light sources

Haematoporphyrin ester (HpE; Paisley Biochemicals, Glasgow, UK), obtained dissolved in saline, was further diluted in saline to a concentration of 100 μg $ml^{-1}$ and stored in aliquots at –20°C. Throughout these experiments HpE was used in a final concentration of 5 μg $ml^{-1}$ of medium. Meta tetra hydroxyphenyl chlorin (mTHPC; Scotia Pharmaceuticals, Guildford, UK) was stored refrigerated as a stock solution of 1 mg $ml^{-1}$ in dimethyl sulphoxide (DMSO) and for experiments was diluted in water and used at a concentration of 0.2 μg $ml^{-1}$. Riboflavin (RB, Sigma) was stored refrigerated as a water solution of 7.5 μg $ml^{-1}$ and used at a concentration of 0.15 μg $ml^{-1}$. The absorption spectrum of RB in the range of visible light was measured in both water solution and in cells preincubated for 24 h with the drug. All photosensitizers and photosensitizer-supplemented cells were carefully handled to avoid light exposure. For HpE and mTHPC sensitization (peak absorbances at 630 nm and 653 nm respectively), a continuous-wave 20-W argon ion-pumped dye laser (Spectra Physics, Hemel Hempstead, UK) was used. The power density of the light at the cell surface was maintained at 20 mW $cm^{-2}$. RB was excited by a non-laser light source emitting continuous wavelength from 400 to 700 nm, and was maintained at the cell surface at 60 mW $cm^{-2}$ (Whitehurst and Moore, 1995). These power densities did not increase the temperature of the medium above ambient.

### Application of PDT and hyperthermia

In all PDT experiments, cells were preincubated for 24 h with a sensitizer-supplemented medium plus serum. All experimental plates were washed once with phosphate-buffered saline (PBS) and the growth medium was replaced with fresh medium just before application of the light treatment. Sensitivity of the 3T6 fibroblasts to the treatments was determined by a clonogenic survival assay and, for the molecular studies, applied at clonogenic equitoxic doses defined as giving 60% cell survival, which were as follows: 1.7 J $cm^{-2}$ for HpE PDT; 1.5 J $cm^{-2}$ for mTHPC PDT; 3 J $cm^{-2}$ for RB PDT and 65 min incubation at 43°C for hyperthermia. The incubation for these in vitro experiments used a Mini Oven Heraeus (DVE, Heraeus, Brentwood, UK). For each series of experiments four types of controls were set: (1) untreated – cells left in the tissue culture incubator; (2) 'low carbon dioxide' – cells removed from the tissue culture incubator for the maximum duration of treatment; (3) 'light-only' – cells exposed to light in the absence of drug; and (4) 'drug-only' – cells incubated with drug but not illuminated.

### Northern hybridization

The total cellular RNA was extracted at 0 (10 min), 3, 6 and 9 h after the treatment application. Cells were washed with saline before extraction using RNAzolB (Biogenesis, Poole, UK), following the manufacturer's instruction with slight modifications: RNA precipitation was carried out at –80°C for 16 h and the final pellet was resuspended in diethyl pyrocarbonate (Sigma) treated water and stored at –80°C. Extracted total RNA was quantified using a Gene Quant II (Pharmacia Biotech, Herts, UK). RNA (20 μg per lane) was separated on 1.2% agarose formaldehyde gel, with ethidium bromide present in the loaded sample and running buffer, and transferred onto a nylon membrane (Hybond-N+, Amersham, Little Chalfont, UK). The successfully transferred RNA was visualized under UV light, and quantities of incorporated RNA on each lane measured as ethidium bromide fluorescence of the 18S and 28S ribosomal bands (Beere et al, 1993). The filter was probed with a 1.5-kb *Eco*RI–*Hin*dIII fragment of murine HSP47 cDNA (Takechi et al, 1992), which was received in plasmid pUC19 as a generous gift from Professor K Nagata

 © Cancer Research Campaign 1997

**Figure 1** Results of analysis by Northern (**A**, **C**, **E** and **G**) and Western hybridization (**B**, **D**, **E** and **F**) of HSP47 expression in 3T6 fibroblasts pretreated with: hyperthermia **A** and **B**; HpE PDT, **C** and **D**; mTHPC PDT, **E** and **F**, and RB PDT, **G** and **H**. Values (means ± 1 s.e.) of HSP47 mRNA and protein expression were calculated as a function of those of untreated controls and represented as '*x*'-fold the control value. Error bars represent inter-experimental variation (*n* = 3–6 repeat experiments). ▧, Low carbon dioxide control; ▩, hyperthermia; ▦ light-only control; □, drug-only control; ■, PDT

© Cancer Research Campaign 1997

**Table 1** Two-way analysis of variance of HSP47 expression (elevated ↑ or reduced ↓), between hyperthermia (HT) and 'low $CO_2$' control and between PDT, 'light-alone' (L) or 'drug alone' controls.

| | HT | | HpE | | mTHPC | | RB | |
|---|---|---|---|---|---|---|---|---|
| Time (h) | N | W | N | W | N | W | N | W |
| 0 | | | | | PDT↓ $P = 0.006$ | | | |
| 3 | | | | | | | | |
| 6 | HT↑ $P = 0.003$ | | | | | L.↑ $P = 0.035$ | PDT↑ $P = 0.0005$ | |
| 9 | | HT↑ $P = 0.005$ | | | | | | PDT↑ $P = 0.009$ |
| 12 | | | | | | | | |

Blank columns for HpE indicate that there were no significant differences at any time. Occupied columns indicate that there were significant differences ($P < 0.05$) between the variable shown and the other(s). Occupied cells show the time at which differences were greatest for Northern (N) and Western (W) hybridization analysis.

(Kyoto University, Japan). The probe was radiolabelled with [α-32P]dCTP (Du Pont, Stevenage, UK), using an oligolabelling method (Oligolabelling Kit, Pharmacia Biotech), and hybridized at 65°C overnight. The negative control was K562 RNA, which was extracted using the same method. The incorporated radioactivity was visualized by autoradiography and quantified on a GS-690 imaging densitometer (Bio-Rad, Hemel Hempstead, UK).

### Western hybridization

Protein was extracted 3, 6, 9 and 12 h after the treatment application. Before extraction cells were washed with PBS, then pelleted and disrupted by sonication. K562 protein was used as the negative control. Protein concentration was determined by the method of Bradford (1976). Protein was separated by 15% one-dimensional sodium dodecyl sulphate–polyacrylamide gel electrophoresis, according to the method of Laemmli (1970), along with biotinylated broad-range SDS standards (Bio-Rad) and transferred on Hybond-C+ membrane (Amersham). The membrane was blocked with 5% bovine serum albumin (BSA) 5% dry milk in PBS–Tween and incubated with anti-HSP47 polyclonal antibody, which was raised in rabbits against rat HSP47 (Stressgen, York, UK), followed by incubation with the secondary anti-rabbit, peroxidase-linked antibody. The HSP47–antibody complex was visualized by enhanced chemiluminescence using Super Signal Substrate Western Blotting (Pierce, Rockford, Illinois, USA) and quantitatively analysed on the GS-690 imaging densitometer. The loading was normalized by the same procedure against actin, using rabbit anti-actin antibody (Biogenesis). The negative control, K562 protein, was used in all experiments.

### RESULTS

The metabolism of HSP47 in 3T6 fibroblasts varied after application of the four different stress conditions. The stress factors were of equivalent toxicity and the variations in HSP47 metabolism were dependent on which stress condition was applied.

Hyperthermia caused an elevation of HSP47 mRNA (Figure 1A and Table 1), and this was observed immediately after its application. The maximum up-regulation was observed at the 6 h time point. At the protein level (Figure 1B), the first up-regulation was detected after 6 h and reached a maximum after 9 h. Lower, although persistent, HSP47 elevation was also observed in 'low carbon dioxide' controls at both transcriptional levels and translational levels (Figure 1A and B).

No significant increase in either HSP47 mRNA (Figure 1C) or protein (Figure 1D) was seen after PDT using HpE.

In 3T6 fibroblasts treated with mTHPC PDT, an absolute increase but a relative decrease of HSP47 mRNA (Figure 1E) and protein (Figure 1F), was detected when compared with the 'drug-only' and 'light-only' controls. The mTHPC alone appeared to induce HSP47 transcription and translation. In addition, a large increase in HSP47 mRNA and protein synthesis was demonstrated after illumination of the cell in the absence of mTHPC, in which the elevation reached its peak at transcriptional level immediately after treatment, and at the protein level after 9 h.

PDT mediated by RB resulted in strong elevation of HSP47, achieving a maximum at the RNA level after 6 h (Figure 1G), and at the protein level 9 h after treatment (Figure 1H). The 'drug-only' control showed slight up-regulation of HSP47, but this was significant only for mRNA. Light-only (no RB) illumination caused enhanced production of HSP47 mRNA and protein 0 h and 6–9 h respectively after the illumination.

The absorption spectrum of 3T6 fibroblast suspensions revealed a strongly marked absorption peak at 656 nm. This observation was not unique to the 3T6 cell line and was also confirmed on other, randomly chosen murine and human fibroblast cell lines (data not shown).

HSP47 elevation was induced by hyperthermia, PDT with RB and light alone (no photosensitizer) illumination in the range 400–700 nm and 653 nm. PDT with HpE and PDT with mTHPC did not have this effect and the latter resulted in a significant relative decrease in HSP47 when compared with the elevated values in the 'light-only' controls.

### DISCUSSION

We have demonstrated that up-regulation of HSP47 by photodynamic injury in 3T6 fibroblasts varies depending on the light/photosensitizer combination. The 3T6 normal murine skin fibroblast cell line is known to biosynthesize procollagen α1(I) and α2(I) chains, secrete them and deposit collagen type I, as seen in extracellular matrix formation. The choice of 3T6 for the purpose of this study enabled us to examine HSP47 in vitro in its in vivo-like functional conditions. The constitutive HSP47 expression level (proportional to rate of collagen type I biosynthesis; Natsume et al, 1994) is high in 3T6 cells. Our choice of 60% cell survival for this initial experiment was driven by a compromise between a reproducible degree of cell kill on the exponential portion of the survival curve and obtaining enough cellular material for analysis. The effects of a high extent of cell kill on HSP47 await further study.

As it was originally detected as a heat shock protein, HSP47 has been reported to be elevated by other factors and physiological conditions (Natsume et al, 1994). All of the HSP47 up-regulation factors described, including hyperthermia, either cause injury to collagen matrix or affect its biosynthesis.

Studies of stress response after PDT are still at an early stage, but inspection of the literature suggests that this aspect of PDT may be complex and heterogeneous. Gomer et al (1988) studied expression of three stress proteins after PDT with porphyrin

 © Cancer Research Campaign 1997

derivatives. GRP78, HSP70 and a 34-kDa protein showed different kinetics, which varied for each of the proteins between smooth muscle, fibroblasts and endothelial cells. Expression of HSP70 was also reported to be increased by Photofrin II-mediated PDT (Fisher et al, 1993), and diminished by aluminium phthalocyanine-mediated PDT (Xue et al, 1995). The experiments showing these two different effects were performed on the same Chinese hamster V79 cell line. Curry and Levy (1993) monitored expression of stress response proteins after PDT, using benzoporphyrin derivative in M1 murine tumour (rhabdomyosarcoma) cells, up to 24 h after treatment. Over time, they detected an elevation of a wide range of proteins, including HSP47. The PDT dose applied for the experiment showing diminished expression of HSP70 resulted in severe morphological changes of M1 cells. The treatment described would probably be harsh enough to disturb the extracellular matrix metabolism, which in a tumour cell line would not necessarily be as stable as in normal cells.

Elevation of HSP47 by hyperthermia on both transcriptional and translational levels has been well studied previously (Nagata et al, 1986), therefore the results obtained here met our expectations and confirmed the applicability of the chosen methods. The detected HSP47 up-regulation in 'low-carbon dioxide' controls was probably induced by change in pH of the medium due to removal of experimental plates from carbon dioxide-enriched tissue culture incubator atmosphere.

The lack of elevation of HSP47 expression after PDT using HpE or even its down-regulation relative to the controls after mTHPC PDT would be consistent with an absence of damage to collagen, although that inference awaits confirmation by on-going studies of collagen type I and its precursor molecules. There is one report presenting evidence for extensive collagen aggregation by $^1O_2$ generated in an HpE-sensitized reaction (Kakehashi et al, 1993). These studies were carried out on collagen in a solution containing HpE at a concentration calculated to be 60 $\mu$g ml$^{-1}$, which is over tenfold greater that the one used by us, and also higher than in clinical applications (Dougherty and Marcus, 1992). Additionally a cell-free environment, unlike a physiological one, lacks factors important for the modulation of active oxygen species, e.g. quenchers of singlet oxygen or superoxide. Curry and Levy (1993) observed HSP47 up-regulation, together with many other stress proteins, using a new PDT sensitizer benzoporphyrin derivative. Their study differed from ours in that (1) the dose they applied reduced cell viability to 30% (6 h after the treatment) and (2) they examined tumour rather than normal cells. We cannot at present rule out that changes in HSP47 simply represent part of a generalized stress response, but if so it is expressed differentially between modalities whose initial 'stress' (in terms of cytotoxicity) was the same.

Where a photosensitizer–light combination is known to damage collagen, e.g. riboflavin plus visible light (Akiba et al, 1994), we observed marked transcriptional and translational up-regulation. Interestingly, a strong increase in HSP47 expression rate was also manifested after illumination by non-laser light alone at the range 400–700 nm and laser wavelength 653 nm. The effect was rapid and was observed immediately after the exposure at the RNA level, whereas the transcriptional up-regulation by PDT with RB and hyperthermia was observed later, i.e. after 3–6 h. The illumination-alone HSP47 elevation is not understood, however our preliminary studies of the 3T6 absorption spectrum (data not shown) suggest the presence of a discrete chromophore with absorption peak at 656 nm.

The molecular events after PDT, in particular, are likely to be complex as at least three 'stresses' may be present: it has been claimed that low levels of light alone have physiological effects (Baxter et al, 1994; Cambier et al, 1996); porphyrins have variable toxicities (Juknatt et al, 1995; Haylett et al, 1996) and PDT is an oxidative stress. The overall outcome includes the possibility of a net down-regulation relative to 'controls' (mTHPC in this paper; Xue et al, 1995).

### ACKNOWLEDGEMENTS

We would like to thank Professor K Nagata (Kyoto University, Japan) for the gift of the plasmid with HSP47 mRNA insert, which enabled us to perform Northern hybridization analysis. We also thank Dr F McNair (PICR, Manchester, UK) for help with raising HSP47 polyclonal antibodies, and Ms A Haylett (PICR) for her valuable practical help and advice. The mTHPC was a kind gift from Scotia Pharmaceuticals. This work was supported by the Association for International Cancer Research (AKV) and the Cancer Research Campaign (JVM).

### REFERENCES

Akiba J, Ueno N and Chakrabarti B (1994) Mechanisms of photo-induced vitreous liquefaction. *Curr Eye Res* **13**: 505–512

Barr H and Bown SG (1992) Normal tissue damage following photodynamic therapy: are there biological advantages? In *Photodynamic Therapy. Basic Principles and Clinical Applications*. Henderson BW and Dougherty TJ (eds), pp. 201–218. Marcel Dekker: New York.

Baxter GD, Walsh DM, Allen JM, Lowe AS and Ball AJ (1994) Effects of low intensity infrared laser irradiation upon conduction in the human median nerve *in vivo*. *Exp Physiol* **79**: 227–234

Beere HM, Morimoto RI and Hickman JA (1993) Investigations of mechanisms of drug-induced changes in gene expression: N-methylformamide-induced changes in synthesis of the M(r) 72,000 constitutive heat shock protein during commitment of HL-60 cells to granulocyte differentiation. *Cancer Res* **53**: 3034–3039

Bown SG (1990) Photodynamic therapy to scientists and clinicians – one world or two? *J Photochem Photobiol B: Biol* **6**: 122

Bradford MM (1976) A rapid and sensitive method for the quantitation of microgram quantities of protein utilising the principle of protein dye binding. *Anal Biochem* **72**: 248–254

Cambier DC, Vanderstraeten GG, Mussen MJ and Van-Der-Spank JT (1996) Low power laser and healing of burns: a preliminary assay. *Plast Reconstr Surg* **97**: 555–558

Cox R and Masson WK (1974) Changes in radiosensitivity during the in vitro growth of diploid human fibroblasts. *Int J Radiat Biol* **26**: 193–196

Curry PM and Levy JG (1993) Stress protein expression in murine tumour cells following photodynamic therapy with benzoporphyrin derivative. *Am Soc Photobiol* **58**: 374–379

Dougherty TJ and Marcus SL (1992) Photodynamic therapy. *Eur J Cancer* **28A**: 1734–1742

Fisher AMR, Ferrario A and Gomer CJ (1993) Adriamycin resistance in Chinese hamster fibroblasts following oxidative stress induced by photodymamic therapy. *Am Soc Photobiol* **58**: 581–588

Foote CS (1991) Definition of type I and type II photosensitised oxidation. *Photochem Photobiol* **54**: 659

Gomer CJ, Ferrario A, Hayashi N, Rucker N, Szirth BC and Murphree L (1988) Molecular, cellular and tissue responses following photodynamic therapy. *Lasers Surg Med* **8**: 450–463

Haylett AK, Forbes E, MacLennan A, Truscott TG and Moore JV (1996) Novel asymetric photosensitisers: an in vitro study. *Cancer Lett* **105**: 187–193

van Hillegersberg R, Kort WG and Wilson PJH (1994) Current status of photodynamic therapy in oncology. *Drugs* **48**: 510–527

Juknatt AA, Kotler ML and Battle AM (1995) High delta-aminolevulinic acid uptake in rat cerebral cortex: effect on porphyrin biosynthesis. *Comp Biochem Physiol C Pharmacol Toxicol Endocrinol* **111**: 143–150

© Cancer Research Campaign 1997

Kakehashi A, Akiba J, Ueno N and Chakrabarti B (1993) Evidence for singlet oxygen-induced cross-links and aggregation of collagen. *Biochem Biophys Res Commun* **196**: 1440–1446

Kato Y, Uchida K and Kawakishi S (1994) Aggregation of collagen exposed to UVA in the presence of riboflavin: a plausible role of tyrosine modification. *Am Soc Photobiol* **59**: 343–349

Laemmli UK (1970) Cleavage of structural proteins during the assembly of the head of the bacteriophage T4. *Nature* **227**: 680–685

Masters A and Bown SG (1992) Interstitial laser hyperthermia. *Semin Surg Oncol* **8**: 242–249

Masuda H, Fukumoto M, Hirayoishi K and Nagata K (1994) Coexpression of the collagen-binding stress protein HSP47 gene and the $\alpha1(I)$ and $\alpha2(I)$ collagen genes in carbon tetrachloride-induced rat liver fibrosis. *J Clin Invest* **94**: 2481–2488

Miyaishi O, Ito Y, Kozaki K, Sato T, Takechi H, Nagata K and Saga S (1995) Age-related attenuation of HSP47 heat response in fibroblasts. *Mech Ageing Dev* **77**: 213–226

Nagata K, Saga S and Yamada KM (1986) A major collagen-binding protein of chick embryo fibroblasts is a novel heat shock protein. *J Cell Biol* **103**: 223–229

Nagata K, Saga S and Yamada KM (1988*a*) Characterisation of a novel transformation-sensitive heat-shock protein (HSP47) that binds to collagen. *Biochem Biophys Res Commun* **153**: 428–434

Nagata K, Hirayoishi K, Obara M and Saga S (1988*b*) Biosynthesis of a novel transformation-sensitive heat-shock protein (HSP47) that binds to collagen. *J Biol Chem* **263**: 8344–8349

Nakai A, Satoh M, Hirayoishi K and Nagata K (1992) Involvement of the stress protein HSP47 in procollagen processing in the endoplasmic reticulum. *J Cell Biol* **117**: 903–914

Natsume T, Koide T, Yokota S, Hirayoishi K and Nagata K (1994) Interactions between collagen-binding stress protein HSP47 and collagen. *J Biol Chem* **269**: 31224–31228

Phillips D (1993/94). Photodynamic therapy. *Science Progress* **77**: 295–316

Sauk JJ, van Kampen CL, Norris K, Foster R and Somerman MJ (1990) Expression of constitutive and inducible HSP70 and HSP47 is enhanced in cells persistently spread on OPN or collagen. *Biochem Biophys Res Commun* **172**: 135–142

Shroff B, Smith T, Norris K, Pileggi R and Sauk JJ (1993) HSP 47 is localised to regions of type I collagen production in developing murine femurs and molars. *Connective Tissue Res* **29**: 273–286

Takechi H, Hirayoishi K, Nakai A, Kudo H, Saga S and Nagata N (1992) Molecular cloning of a mouse 47 kDa heat-shock protein (HSP47), a collagen-binding stress protein, and its expression during the differentiation of F9 teratocarcinoma cells. *Eur J Biochem* **206**: 323–329

Thomsen S (1991) Pathologic analysis of photothermal and photomechanical effects of laser-tissue interactions. *Photochem Photobiol* **53**: 825–835

Whitehurst C and Moore JV (1995) Development of an alternative light source to lasers for biomedical applications. *Biomed Optoelectron Clin Chem Biotechn* **2629**: 291–298

Xue L, Agarwal ML and Varnes ME (1995) Elevation of GRP-78 and loss of HSP70 following photodynamic therapy treatment of V79 cells sensitization by nigericin. *Photochem Photobiol* **62**: 135–143

© Cancer Research Campaign 1997